# Epidemiology of Cerebral Venous Sinus Thrombosis: 2021-2023

**DOI:** 10.7759/cureus.93654

**Published:** 2025-10-01

**Authors:** Avi Ruderman, Andrew Hogan, Chad Epley, Daniel M Courtney, Brian Milman

**Affiliations:** 1 Emergency Medicine, University of Texas Southwestern Medical Center, Dallas, USA

**Keywords:** cerebral venous sinus thrombosis, cvst, emergency medicine, neurovascular disorders, stroke

## Abstract

Cerebral venous sinus thrombosis (CVST) is a high-risk, low-prevalence disease. Presentations can be variable, and diagnosis often requires magnetic resonance venography, making diagnosis challenging. To date, no studies have focused on patients presenting to the emergency department (ED) who were ultimately diagnosed with CVST. We conducted a cross-sectional study using Cosmos (Epic Systems Corporation), a large de-identified electronic health record database. Our objective was to describe the ED encounter rate, demographic characteristics, clinical features, medications, and outcomes of adult patients with CVST between 2021 and 2023. Our age distribution indicates that CVST occurs more frequently in older adults than previously thought. Headache was the most common chief complaint. Co-presentation with head injury and malignancy was also frequent. Most patients underwent some form of neuroimaging and received anticoagulation. In this large, nationally representative ED cohort, CVST was rare; however, prospective studies are needed to better understand the risk factors, diagnostic approaches, and medical management of CVST in patients presenting to the ED.

## Introduction

Cerebral venous sinus thrombosis (CVST) is a high-risk but low-prevalence disease with frequent delays in diagnosis [1]. High-risk, low-prevalence diseases are particularly challenging in the emergency department (ED) due to their variable presentations, limited findings on common diagnostic modalities, and the need for rapid diagnostic and treatment decisions in a resource-limited, high-acuity setting. The largest prospective case series published on this condition is from the International Study on Cerebral Vein and Dural Sinus Thrombosis (ISCVT) [2]. ISCVT was a prospective multinational observational study conducted between 1998 and 2001 and included 624 adults from 89 centers in 21 countries. In this study, there was a 3:1 ratio of females to males with a median age of 37. In contrast, a recent retrospective study evaluating over 57,000 patients with CVST between 2005 and 2016 in the United States (US) reported a 2:1 ratio of females to males with a significantly higher mean age of 45 [3]. Another study evaluating the incidence of CVST in British Columbia, Canada, over a similar time period reported a ratio of 0.8:1 female to male with an average age of 51 [4]. The reported incidence rates of CVST range from two to 20 cases per million per year [3,5]. Much of the variance in study results can be attributed to differences in sampling methods, as well as evolving imaging technology and clinicians' testing thresholds. What remains consistent, however, is the low incidence of disease detection. The discrepancies between the findings of the above studies could be reconciled with a large nationwide sample, allowing for measurement of prevalence as well as patient-level information. This would provide a better picture of what CVST clinically looks like in the ED setting. 

Increasingly available large national databases, consisting of aggregated electronic health record (EHR) data, allow for current and representative investigations into the epidemiology of CVST [6]. The primary objective of this study was to report on the national epidemiological data and available clinical data for this condition in EDs in the US between 2021 and 2023. 

## Materials and methods

We conducted a cross-sectional study of patients who presented to US EDs from 2021 to 2023 and were diagnosed with CVST. Data were obtained using Cosmos (Epic Systems Corporation), a large, de-identified EHR database that aggregates data from numerous healthcare organizations that use the Epic EHR. Cosmos provides a representative sample of patients who seek healthcare and have a similar age, race, ethnicity, coverage, and social vulnerability index distribution to the US Census. At the time of our Cosmos query, Cosmos included 300 million unique patients from over 1,700 hospitals across all 50 states, Washington D.C., Canada, Lebanon, and Saudi Arabia. 

Our study included US ED encounters from January 1, 2021, to December 31, 2023. We included patients aged 18 years or older at the time of visit with an admitting diagnosis, billed admitting diagnosis, or billed final diagnosis of CVST using the Systematized Nomenclature of Medicine - Clinical Terms (SNOMED CT) concept ID 192759008 (cerebral venous sinus thrombosis). Child concepts within this concept hierarchy are included in supplemental information. 

For each eligible encounter, we extracted the following demographic, clinical, and outcome variables: age, legal sex, race per Office of Management and Budget defined categories, ethnic group, body mass index (BMI), chief complaint at the time of ED arrival, pregnancy status based on pregnancy episodes in Epic, comorbidities (polycythemia vera, sickle cell anemia, systemic lupus erythematosus, other coagulation disorders) using International Classification of Disease (ICD)-10 codes, co-presenting diagnoses (sepsis, head injury, and malignancy) using SNOMED CT concepts, hospital-administered medications, imaging performed using Current Procedural Terminology codes, ED disposition, hospital disposition, in-hospital mortality, and 30-day readmission. We described population characteristics using proportions. 

## Results

During the three-year study period, 99,722,660 total ED encounters were recorded. Of these, 4,307 (0.004%) included a CVST diagnosis, corresponding to a rate of 43 cases per one million ED encounters. The study population demographics are reported in Table 1.

**Table 1:** Demographics of the study population CVST: cerebral venous sinus thrombosis; ED: emergency department; BMI: body mass index

Demographic		CVST, N (%)	Total ED population, N (%)
Age	Young adult (18-24)	392 (9.1)	10,962,633 (11.0)
	Adult (25-44)	1,351 (31.4)	31,899,362 (32.0)
	Middle-aged (45-64)	1,374 (31.9)	28,312,101 (28.4)
	Aged (65-79)	864 (20.1)	18,789,783 (18.8)
	80 and over	326 (7.6)	9,758,778 (9.8)
Legal sex	Male	1,698 (39.4)	44,143,787 (44.3)
	Female	2,609 (60.6)	55,556,271 (55.7)
Race	White communities	3,103 (72.0)	67,981,502 (68.2)
	Black communities	809 (18.8)	24,817,587 (24.9)
	Asian	198 (4.6)	2,413,636 (2.4)
	American Indian or Alaska native	64 (1.5)	1,551,073 (1.6)
	Other	678 (15.7)	15,562,999 (15.6)
Ethnic group	Not Hispanic or Latino	3,631 (84.3)	84,149,822 (84.4)
	Hispanic or Latino	507 (11.8)	11,209,430 (11.2)
	None of the above	169 (3.9)	4,363,408 (4.4)
BMI	Underweight	177 (4.1)	2,702,702 (2.7)
	Normal	1,224 (28.4)	23,499,108 (23.6)
	Overweight	1,422 (33.0)	24,928,690 (25.0)
	Obese	2,029 (47.1)	36,069,772 (36.2)
Insurance coverage	Commercial	2,081 (48.3)	44,071,403 (44.2)
	Medicare	684 (15.9)	15,658,971 (15.7)
	Other insurance	370 (8.6)	9,032,204 (9.1)
	None of the above	1,172 (27.2)	30,960,082 (31.0)

For patients diagnosed with CVST, the most common chief complaints were headache (1,252; 29.1%), altered mental status (273; 6.3%), dizziness (231; 5.4%), and eye problems (180; 4.2%). The frequency of additional chief complaints is shown in Table 2. 

**Table 2:** Frequency of chief complaint CVST: cerebral venous sinus thrombosis; ED: emergency department; AMS: altered mental status

Chief complaint	CVST, N (%)	Total ED population, N (%)
Headache	1,252 (29.1)	3,312,235 (3.3)
AMS	273 (6.3)	1,732,869 (1.7)
Dizziness	231 (5.4)	2,740,522 (2.7)
Eye problem	180 (4.2)	553,811 (0.6)
Stroke	161 (3.7)	235,828 (0.2)
Vomiting	155 (3.6)	3,579,925 (3.6)
Seizures	150 (3.5)	737,591 (0.7)
Numbness	136 (3.2)	605,407 (0.6)
Weakness-generalized	136 (3.2)	2,094,298 (2.1)
Fall	125 (2.9)	4,784,418 (4.8)
None of the above	1,789 (41.5)	80,707,377 (80.9)

In patients with CVST, 1,351 (31.4%) co-presented with head injury, 922 (21.4%) with malignancy, and 255 (5.9%) with sepsis. As highlighted in Table 3, a notable minority of patients had conditions that predisposed them to hypercoagulability. 

**Table 3:** Rates of hypercoagulable conditions ^*^Indicates a group of ICD-10 codes. CVST: cerebral venous sinus thrombosis; ED: emergency department

Condition	CVST, N (%)	Total ED population, N (%)
Other coagulation disorders (D68.^*^)	740 (17.2)	938,807 (0.9)
COVID-19 infection	217 (5.0)	5,074,688 (5.1)
Systemic lupus erythematosus	60 (1.4)	384,555 (0.4)
Pregnancy	59 (1.4)	2,270,431 (2.3)
Sickle cell anemia	40 (0.9)	355,555 (0.4)
Polycythemia vera	25 (0.6)	32,549 (0.03)

The most common imaging modalities used during evaluation of the CVST population were computed tomography (CT) of the head with or without contrast (2,967; 68.9%), magnetic resonance (MR) of the brain with or without contrast (2,710; 62.9%), CT angiography of the head (2,365; 54.9%), MR angiography of the head (1,781; 41.4%), and CT angiography of the neck (1,467; 34.1%). Patient-level radiology reports are unavailable in Cosmos. 

The CVST population received medications from varied pharmacological classes (Table 4). Anticoagulants were commonly used in the CVST population. Among these, heparin products were most frequently administered, with 3,189 (74.0%) patients receiving enoxaparin and/or unfractionated heparin (33.8% and 57.1%, respectively). The most prevalent oral anticoagulants used in this population were apixaban (1,268; 29.4%), warfarin (455; 10.6%), and rivaroxaban (251; 5.8%). Patients received thrombolytic medications in 140 (3.3%) CVST cases (alteplase in 3.0% and tenecteplase in 0.3%). Benzodiazepines were administered to 1,626 (37.8%) patients, and 939 (21.8%) patients received levetiracetam. Osmotic therapies were administered in 247 (5.7%) cases, with hypertonic saline being used in 177 (4.1%) and mannitol in 70 (1.6%).

**Table 4:** Medications administered to CVST patients CVST: cerebral venous sinus thrombosis

Medication class	Medication name	N (%)
Anticoagulants	heparin	2,461 (57.1)
	enoxaparin	1,454 (33.8)
	apixaban	1,268 (29.4)
	warfarin	455 (10.6)
	rivaroxaban	251 (5.8)
	dabigatran	197 (4.6)
Thrombolytics	alteplase	129 (3.0)
	tenecteplase	11 (0.3)
Anticonvulsants	benzodiazepines	1,626 (37.8)
	levetiracetam	939 (21.8)
	propofol	224 (5.2)
Osmotic agents	3% sodium chloride	177 (4.1)
	mannitol	70 (1.6)

Among patients diagnosed with CVST, 3,434 (79.7%) were admitted or placed into observation from the ED, an additional 211 (4.9%) were transferred to another hospital from the ED, and 363 (8.4%) were discharged from the ED. The 30-day readmission rate for the CVST population was 6.8%. The median length of stay for admissions was four days (IQR 3-8). Additional hospital dispositions can be seen in Table 5. 

**Table 5:** Disposition of CVST patients CVST: cerebral venous sinus thrombosis

Disposition		N (%)
ED disposition	Admit	3,185 (74.0)
	Observation	249 (5.8)
	Discharge	363 (8.4)
	Transfer	211 (4.9)
Hospital disposition	Discharged to home or self-care	2,800 (65.0)
	Discharged to home under the care of home health	347 (8.1)
	Discharged/transferred to short-term hospital for inpatient care	235 (5.5)
	Discharged/transferred to skilled nursing facility	207 (4.8)
	Discharged/transferred to inpatient rehab facility	181 (4.2)
	Left against medical advice	57 (1.3)
	Hospice-medical facility	49 (1.1)
	Hospice-home	44 (1.0)
	Discharged/transferred to another type of healthcare facility	49 (1.1)
	None of the above	199 (4.6)

## Discussion

CVST is a rare type of stroke with significant morbidity and difficulty in diagnosis due to its prevalence, as well as the necessity of less common and more expensive radiologic procedures for diagnosis [7]. This study is the largest cross-sectional evaluation of epidemiologic data associated with CVST to date, and the only study focused on ED encounters. It is also a more contemporary data set than any other published CVST studies.

Our study reports an encounter rate of 43 cases per one million ED encounters in the US, confirming the rarity of this condition. This rate cannot be directly compared to incidence rates using a general population but is within an order of magnitude of published population-based incidence rates [8-10]. The incidence rate in the ED population would be expected to be lower than the overall incidence rate, as some cases of CVST are diagnosed in the outpatient setting or by autopsy. Additionally, many patients in a given population present to the ED more than once per year, though our methodology is expected to capture some cases that would be missed using hospital admission or discharge diagnoses (e.g., patients who expired in the ED, were discharged, or were transferred). The ED encounter rate may overstate the true incidence by including repeat ED visits by the same patient for which this diagnosis was included. Additionally, our study describes the first CVST cohort including patients who presented to the ED during the COVID-19 pandemic. The rate of concurrent COVID-19 infection and CVST within the cohort is reported above, although the full impact of COVID-19 infection on the incidence of CVST compared to prior years remains unclear [11]. 

The sex ratio of our CVST cohort was 1.5:1 female to male. Prior studies have conflicted on the sex ratio, but our finding is slightly lower than the other large US-based cohort [2-4,12]. The lower ratio from our analysis could reflect changing ratios over time, as the prior study used a data set from 2006 to 2016. Alternatively, the greater geographic breadth of this study may better represent the average sex ratio in the US compared to the two individual states included in the prior study. It is also possible that the sex ratio was affected by the COVID-19 pandemic. Nevertheless, our CVST sex ratio is similar to that of the overall ED encounter cohort, which may suggest that female sex is less associated with CVST than previously thought. 

The average age of patients with CVST has been reported to be notably lower than that of patients with ischemic stroke [13]. Our study replicated this finding, observing 1,743 (40%) CVST patients between the ages of 18 and 44, and 3,117 (72%) between the ages of 18 and 64. The mean age for patients with CVST was 49 for females and 54 for males, compared to the median age of 71 reported for ischemic stroke in the US [14]. While this distribution skews younger than ischemic stroke, it is notable that 1,190 (28%) of the patients in our cohort were 65 or older. The rate of CVST was lowest for patients aged 18-24 and ≥80 (36 and 33 cases per million ED encounters, respectively). The rate was highest among patients aged 45-64 and 65-79 (49 and 46 cases per million ED encounters). These findings challenge the notion that CVST is primarily a disease of the young and suggest the condition may primarily affect those in middle to older age. Given the limitations of our data abstraction method, this finding requires further investigation for confirmation.

Our data found an encounter rate for Asian patients (82 per one million ED encounters) approximately twice that of other racial groups (White communities, Black communities, American Indian/Alaska Native, or other). This increased signal in the Asian population has been noted previously and deserves further investigation to better elucidate any association [15]. 

Headache was the most common chief complaint in 1,252 (29%) of the CVST cohort (Table 2). No other chief complaint exceeded 7%. The rate of headache is significantly lower than the 70%-90% reported in previous studies [16]. Importantly, 1,789 (41.5%) of chief complaints were either symptoms not commonly associated with CVST or free-text entries that could not be abstracted using our methodology. As a result, the frequency of individual chief complaints such as headache, altered mental status, or confusion is likely underrepresented. 

Patients with CVST co-presented with head injury in 1,351 (31%) cases. This may indicate that falls or seizures may have been more frequent than our chief complaint data reports. Malignancy was listed as a co-presentation in 922 (21%) cases, which potentially reflects the hypercoagulable effects of cancer. Malignancy was far more commonly associated with CVST than any of the other hypercoagulable conditions we investigated. Sepsis was coded as a co-presentation in 255 (6%) cases, and it is unclear whether this represented true infection or was included due to nonspecific findings such as leukocytosis and tachycardia. 

Among patients with CVST, 117 (2.7%) were labeled with a diagnosis code of "CVST during pregnancy," which is slightly more than the overall pregnancy rate of 2.3% within the general ED population. Because some pregnant patients with CVST may not have been labeled with a diagnosis code of "CVST in pregnancy," it is possible that the pregnancy rate in the CVST population could have been underreported. Based on these results, in this study group, a few patients with CVST were pregnant. Prior studies have shown higher incidence rates in females of childbearing age but have not distinguished between pregnant and non-pregnant women. Those findings might have been due to alternative risk factors such as the use of oral contraceptive pills. An additional 117 (2.7%) CVST patients were specifically coded as "CVST in puerperium," although it was not possible to determine the percentage of recently postpartum patients in the overall ED population using our methods. Assuming that the percentage of patients in the post-partum period is a fraction of the percentage of patients who are currently pregnant, the percentage of CVST patients in the post-partum period appears higher than expected, and the post-partum period may represent a significant risk factor for CVST worthy of future investigation. We evaluated several conditions that are generally associated with hypercoagulability: COVID-19, systemic lupus erythematosus, sickle cell anemia, and polycythemia vera [17]. Aside from COVID-19 (5.0% of the CVST cohort compared to 5.1% of the ED population), each of these conditions showed an increased rate among CVST patients while still representing a very small proportion of the cohort (Table 3). Seven-hundred-forty (17.2%) patients were coded as having “other coagulation disorders” compared to 0.9% of the ED population. These cases were identified using all D68 ICD-10 codes. In our population, the most common diagnoses that made up this group were other primary thrombophilia (D68.59), other thrombophilia (D68.69), coagulation defect, unspecified (D68.9), activated protein C resistance (D68.51), antiphospholipid syndrome (D68.61), lupus anticoagulant syndrome (D68.62), hemorrhagic disorder due to extrinsic circulating anticoagulants (D68.32), and prothrombin gene mutation (D68.52). All ICD-10 codes from our patient population that fell under other coagulation defects (D68.*) are included in the supplementary material. In patients who were coded as D68.59, D68.69, and D68.9, it is unclear if these disorders represented an underlying coagulation defect or whether these patients were labeled with this code solely by virtue of having suffered a CVST. In our population, the rates of CVST were similar in patients with and without diagnosis of COVID-19. 

Our study demonstrated substantial heterogeneity in imaging studies. Most patients received a CT of the head (2,967; 69%) or MRI of the brain (2,710; 63%) or CT angiography of the head (2,365; 55%). Fewer patients underwent MR angiography of the head (1,781; 41%) despite that being the test of choice for diagnosing CVST [18]. Of note, it was not possible to use our methodology to determine whether arterial- or venous-phase imaging protocols were used for the CT and MR angiography studies, limiting our ability to draw conclusions from this data.

Systemic anticoagulation is the standard of care for CVST, and 3,575 (83%) of the patients in the CVST cohort received some form of anticoagulant medication. Specifically, 1,716 (40%) received a direct oral anticoagulant (DOAC), 3,189 (74%) received either heparin or enoxaparin, and 455 (10.6%) received warfarin. Our data did not allow for conclusions about why a particular agent was chosen, and these decisions may have been affected by factors such as provider discretion, medication cost, patient ability to take oral medications, or case-by-case need for potential rapid reversal of anticoagulation. Only 3,575 (83.0%) of the 4,307 CVST patients received any anticoagulation during their hospital course. Whether the remaining 732 (17.0%) were discharged with a plan to initiate anticoagulant medications after discharge, were truly discharged without any anticoagulation, or expired prior to receiving an agent other than unfractionated heparin could not be determined from our data. 

Systemic thrombolysis has not been endorsed as a standard treatment for CVST, yet 140 (3.3%) patients in our cohort received either alteplase or tenecteplase. This finding may be explained by administration of thrombolytics in cases of CVST that were initially diagnosed as acute ischemic stroke or in cases with severe morbidity. 

The overall mortality rate in our study was 3.2% (95% CI: 2.7%-3.8%), similar to previously reported rates [2]. Most admitted patients (2,800; 65%) were discharged home from the hospital without assistance, while 1,063 (24.7%) were discharged to either home health, inpatient rehabilitation, a skilled nursing facility, a long-term care facility, or hospice care. These numbers illustrate the significant morbidity associated with CVST. 

Limitations

This study has several limitations primarily related to the data abstraction method. The data represent ED encounters and not specific patients. We therefore measured an ED encounter rate rather than true incidence. Our abstraction method included patients diagnosed with CVST after admission, who may not have initially presented with this condition. Such patients include those developing CVST after a neurosurgical intervention or while recovering from a traumatic brain injury. Additionally, we had no way to verify that patients coded as having CVST in fact had the condition, and our data may include diagnostic misclassification or administrative coding errors, which could bias the encounter rate and associated clinical characteristics. Several categories of data were not well reported, such as BMI, complicating the ability to stratify patients by weight. The current version of Cosmos does not allow for the abstraction of pregnancy status with a high degree of confidence due to the numerous ways that pregnancy can be determined (e.g., patient report, urine testing, serum testing, or imaging study). Medications administered could not be separated by indication or time point, and so patients may have received a benzodiazepine for seizure, to facilitate radiographic imaging, for general anxiety, or for another reason during their hospitalization. Chief complaints, radiographic imaging studies, and comorbidities could not be reliably abstracted. Cosmos is unable to abstract chief complaints that were entered as free-text rather than selecting a standard option, likely undercounting the rates of each complaint. For example, a chief complaint of “headache and vomiting” would have been coded as neither in our data. Forty-two percent of chief complaints were either not commonly associated with CVST syndromes or were free-text entries. CT venograms and CT angiograms are coded the same and so cannot be differentiated. Similarly, MR venograms and MR angiograms cannot be differentiated in our data. Each of these limitations significantly limited the strength of our associated conclusions.

## Conclusions

In this study, we used Epic Cosmos, a nationally representative database of electronic health record data from over 1,700 hospitals, to evaluate the ED encounter rate and characteristics of patients presenting to US EDs with CVST. We present a large, nationally representative cohort over a contemporaneous time period and provide new insights into a rare and poorly understood disease. Our results demonstrate the rarity of the condition, variability in diagnostic and therapeutic approaches, and challenge historical data on age- and sex-based incidence. Notably, this study is the first to document CVST rates during the COVID-19 pandemic, when infection with the virus was believed to confer increased thrombotic risk. Based on our findings, further studies are needed to prospectively describe the adult population that presents to the ED and is diagnosed with CVST.

## Appendices

Systematized Nomenclature of Medicine-Clinical Terms (SNOMED CT) concept ID 192759008 (cerebral venous sinus thrombosis) child concepts:

Cerebral venous thrombosis of the sigmoid sinus (disorder) Thrombophlebitis of the sigmoid sinus (disorder) Septic thrombophlebitis of the sigmoid sinus (disorder) Cerebral venous thrombosis of the straight sinus (disorder) Neonatal noninfectious cerebral venous sinus thrombosis (disorder) Neonatal thrombosis of the cerebral venous sinus (disorder) Non-pyogenic venous sinus thrombosis (disorder) Non-pyogenic thrombosis of the intracranial venous sinus (disorder) Septic thrombophlebitis of the sagittal sinus (disorder) Thrombophlebitis of the lateral venous sinus (disorder) Septic thrombophlebitis of the lateral sinus (disorder) Thrombosis of the basilar sinus (disorder) Thrombophlebitis of the basilar sinus (disorder) Thrombosis of the cavernous venous sinus (disorder) Thrombophlebitis of the cavernous sinus (disorder) Septic thrombophlebitis of the cavernous sinus (disorder) Thrombosis of the cerebral venous sinus due to and following a surgical procedure (disorder) Thrombosis of the inferior sagittal sinus (disorder) Thrombophlebitis of the inferior sagittal sinus (disorder) Thrombosis of lateral venous sinus (disorder) Thrombosis of the superior sagittal sinus (disorder) Thrombophlebitis of the superior sagittal sinus (disorder) Thrombosis of torcular Herophili (disorder) Thrombophlebitis of torcular Herophili (disorder) Thrombosis of the transverse sinus (disorder) Thrombus of the dural sinus in pregnancy (disorder) Thrombus of the dural sinus in puerperium (disorder) 

International Classification of Diseases-10 codes grouped in other coagulation disorders (D68.*):

D68.59, D68.69, D68.9, D68.51, D68.61, D68.62, D68.32, D68.52, D68.8, D68.2, D68.4, D68.69, D68.311, D68.5, D68.0, D68.09, D68.04, D68.1, D68.01, D68.020, D68.021, D68.022, D68.023, D68.03, D68.00, D68.029, D68.312, D68.3, D68.31, D68.318
